# Neuromodulation and the mind-brain relation

**DOI:** 10.3389/fnint.2015.00022

**Published:** 2015-03-23

**Authors:** Walter Glannon

**Affiliations:** Department of Philosophy/Arts, University of CalgaryCalgary, Canada

**Keywords:** brain-mind interaction, dualism, neuromodulation, non-reductive materialism, psychiatric disorders, reductive materialism

Many investigators now consider psychiatric disorders to be neural circuit disorders (Lozano and Lipsman, 2013). Cognitive, emotional and volitional symptoms of major depression, generalized anxiety, obsessive-compulsive disorder and other conditions are traceable to dysfunction in critical nodes of circuits in cortical, limbic and subcortical pathways of the brain. Guided by functional imaging, the ability of deep-brain stimulation (DBS) to probe and modulate specific circuits in real-time has elucidated the pathogenesis of these disorders. DBS has validated the neurobiological underpinning of normal and abnormal states of mind. This and other developments in clinical neuroscience discredit a dualist theory of mind and brain that takes psychological properties to be conceptually distinct from and capable of functioning independently of neural properties. Instead, they support a materialist theory of mind that explains mental phenomena in terms of their neural correlates.

A materialist theory of mind suggests that mental illnesses are just diseases of the brain (Fuchs, 2012). This idea is generally consistent with the aim of the Research Domain Criteria (RDoC) initiated by the US National Institutes of Mental Health (NIMH), which is to identify brain mechanisms that can explain the causes of psychiatric disorders and predict treatment responses and outcomes (Insel et al., 2010; Casey et al., 2013). There are, however, reductive and non-reductive versions of materialism (Baker, 2009). According to reductive materialism, phenomena at one level can be completely explained in terms of more basic elements at a different level. On this view, normal and abnormal mental states can be completely explained in terms of brain function and dysfunction. According to non-reductive materialism, the brain necessarily generates and sustains mental states but cannot account for all of their properties. By themselves, probing and modulating neural circuits of people with psychiatric disorders fail to capture how the content and phenomenology of the mind can affect the brain in how these disorders develop and respond to treatment.

Psychiatric disorders are multifactorial disorders resulting from interaction among genes, neurons, immune and endocrine systems and the affected person's psychological response to the natural, social and cultural environment. Describing these disorders at a brain-systems level is necessary but not sufficient for understanding their etiology or how they can be controlled through different types of neuromodulation. It is not dysfunctional neural circuits but people who have these disorders. Persons are constituted by their brains but are not identical to them. The conscious and unconscious mental states that emerge from the brain and define persons are influenced by a dynamic and interacting set of factors both inside and outside of the brain. Mind and brain are shaped by the fact that persons are embodied and embedded in different environments. Our brains alone do not determine everything about who we are and how we experience the world (Churchland, 2013). These considerations suggest that non-reductive materialism is a more plausible theory than reductive materialism for explaining the mind-brain relation in psychiatry and a more helpful model for diagnosing and treating psychiatric disorders.

Neuropsychiatrist Todd Feinberg's conception of the mind as a process emerging from the brain in a nested hierarchy supports non-reductive materialism as a theoretical basis of the mind-brain relation (Feinberg, 2001, pp. 129–131). Higher-level processes associated with conscious and unconscious mental states are compositionally dependent on, or nested within, lower-level processes associated with circuits in the brain. Feinberg's idea of constraint can be used to explain how interacting neural and mental processes promote homeostasis within an organism and its adaptability to the external world. Constraint refers to the control that one level of a system exerts over another level of the same system. The “system” at issue is a human organism, and the relevant “levels” are brain and mind. Constraint operates in both bottom-up and top-down directions as brain and mind mutually influence each other in a series of re-entrant loops. Neural functions constrain mental states to ensure that they accurately interpret information from the environment. Mental states constrain neural circuits to ensure that they are neither underactive nor overactive. Beliefs with heightened emotional content can over-activate the limbic fear system, disable cortical constraint on this system and lead to depression, anxiety or panic disorders. Disabled constraints on belief content from dysfunctional auditory and prefrontal cortices can result in the hallucinations and delusions in the positive subtype of schizophrenia.

Proponents of non-reductive materialism hold that mental properties are part of the material world. They also hold that mental properties can be causally efficacious without being reducible to material properties. Critics of this position argue that if mental events are not reducible to physical events, then they are epiphenomenal (Kim, 1998, p. 81). Mental events are the effects of material or physical causes but cannot cause any material or physical events to occur. If mental processes associated with beliefs, desires and emotions are not reducible to their neural correlates and are epiphenomenal, then presumably they do not influence the etiology of psychiatric disorders or patients' responses to therapies.

But there are many examples in psychiatry where mental states are causally efficacious in disrupting and modulating neural pathways. Persistent psychological stress can cause a cascade of adverse biochemical events in the brain and body, including hyperactivation of the amygdala fear system and dysregulation of the hypothalamic-pituitary-adrenal (HPA) axis and sympathetic nervous system. This can disrupt frontal-limbic connectivity mediating cognitive and affective processing and result in impaired cognition and mood. The contents of a person's beliefs and emotions may play a causal role in the pathophysiology of this disorder. In obsessive-compulsive disorder, excessive conscious reflection on motor tasks ordinarily performed as a matter of course can have a similar disruptive effect on frontal-limbic-striatal pathways and impair sensorimotor and cognitive functions (Melloni et al., 2012; Figee et al., 2013). Yet mental states can be part of a therapeutic process as well. In cognitive behavior therapy (CBT), patients with depression can be trained to reframe their beliefs and emotions in a way that can re-wire some regions of the brain and result in significant improvement in depressive symptoms. Studies have shown that CBT can modulate function in specific sites in limbic and cortical regions mediating mood and cognition (Fuchs, 2004; Goldapple et al., 2004). Disruptive bottom-up effects on mental functions by dysregulated neural functions can be reversed to some degree by top-down modulating effects of this therapy on these functions.

Neurofeedback (NFB) is another example of how mental states can modulate brain activity. With this technique, participants can be trained to down-regulate brain hyperactivity through their cognitive and emotional responses to the sensory feedback of neural function they receive from EEG or fMRI (Linden et al., 2012; Linden, 2014, chapter 3). This type of self-regulation can restore some degree of control of thought and behavior for those who successfully perform the technique and relieve symptoms associated with attention deficit-hyperactivity disorder (ADHD), anxiety, depression, posttraumatic stress disorder (PTSD) and other conditions. It may also be possible for depressed subjects with anhedonia and avolition associated with an underactive nucleus accumbens in the reward system to use NFB to up-regulate activity in this region and improve motivation (Linden, 2014, chapter 3). NFB demonstrates that participants can induce changes in their brains through their own mental states without having to rely on psychoactive drugs or devices implanted and stimulated in specific neural circuits. Moreover, the fact that the cognitive and emotional responses that induce these changes depend on indices of brain activity fed back to the subject shows that mind and brain are not independent but interdependent and interacting processes necessary for flexible and adaptive behavior. It highlights the erroneous assumption that non-reductive materialism implies dualism between mental properties and neural properties and that the first cannot influence the second. It is because of interaction between brain and mind in NFB that this technique can produce its therapeutic effects. Indeed, some investigators describe NFB as “a holistic approach that overcomes bio-psychological dualisms” (Linden et al., 2012, p. 8).

Psychological factors are also significant in brain-computer interfaces (BCIs) used as a form of neurofeedback for neurological disorders. These systems may use scalp-based electrodes to record EEG or a microelectrode array implanted in the motor cortex. More and less invasive forms of BCIs are designed to enable subjects paralyzed from spinal cord or traumatic brain injuries to bypass the site of injury and translate signals from the motor cortex through a computer into actions such as moving a cursor or robotic arm. In addition, BCIs have been studied to determine whether individuals with complete locked-in syndrome can communicate when they are unable to do this verbally or gesturally by activating signals in brain regions mediating semantic processing. Subjects have to be trained to perform these neural and mental acts in manipulating the interface, and there is considerable variation among them in the capacity to be trained. Success in learning how to use the system and activate and translate signals in the motor cortex depends on operant conditioning, which requires sustained motivation, attention and persistence. Not all locked-in subjects have the requisite degree of these psychological capacities to manipulate the interface. One explanation for the failure of researchers and practitioners to train these individuals to communicate with a BCI is that the complete loss of control from paralysis undermines the motivational basis for operant conditioning (Birbaumer et al., 2008, 2014; Linden, 2014, p. 22). They may experience not only physical fatigue but also mental fatigue in repeatedly attempting and failing to activate the cortical regions necessary for communication. Theoretically, though, participants who are sufficiently motivated and have the necessary cognitive capacity could be trained to translate signals in these regions into actions that realized their intentions. Psychological properties of the participant play a causal role in the success or failure in using the technique to induce the desired brain responses. In both NFB and BCIs, the role of the trainer in enabling the participant to exercise the critical mental capacities and induce changes in the brain is one aspect of environmental influence on these capacities and changes.

In treatment-refractory depression and obsessive-compulsive disorder, DBS can modulate dysfunctional circuits enough to make them amenable to CBT. As in NFB, this underscores the complementarity of brain-based and mind-based techniques in controlling symptoms in these disorders. Mind-brain dualism would conceive of these techniques as unrelated or even incompatible. The reductionist view that everything about the mind can be explained by appeal to neural circuits and that mental states are epiphenomenal would also fail to appreciate the causal efficacy of beliefs and other cognitive states on neural function. Dualist and reductive materialist models of the mind and brain both fail to recognize the salutary effects of combined neural and psychological therapies for these disorders. It is thus mistaken to assume that non-reductive materialism in clinical neuroscience implies that mental states have no influence on the brain.

Rather than focusing exclusively on neural mechanisms, a holistic model of psychiatric disorders that explains them in terms of interaction among genes, neurons, mental states, the body and the environment is more helpful in understanding them. The success or failure of different forms of neuromodulation for these disorders demonstrates that interdependent mental and neural processes influence the extent to which they can be controlled. These considerations support a non-reductive materialist model of the mind-brain relation in psychiatry. This rejects the reductionist view that mental illnesses are just diseases of the brain and that how we experience the world is completely determined by neural structure and function. Theoretical models such as the RDoC and techniques such as DBS alone may be too limited to provide an adequate account of mental health and mental illness. They are components of a broader set of factors that shape mind-brain interaction and need to be included in diagnosing and treating psychiatric disorders.

## Conflict of interest statement

The author declares that the research was conducted in the absence of any commercial or financial relationships that could be construed as a potential conflict of interest.
